# Predictive score for estimating cancer after venous thromboembolism: a cohort study

**DOI:** 10.1186/1471-2407-13-352

**Published:** 2013-07-22

**Authors:** Bruno L Ferreyro, Federico Angriman, Diego Giunta, María Lourdes Posadas-Martínez, Fernando Vazquez, Fernán Gonzalez Bernaldo De Quirós, Andre C K B Amaral, Damon C Scales

**Affiliations:** 1 Internal Medicine Department, Hospital Italiano de Buenos Aires, Buenos Aires University, Buenos Aires, Argentina; 2 Internal Medicine Research Unit, Hospital Italiano de Buenos Aires, Buenos Aires, Argentina; 3 Internal Medicine Department, Hospital Italiano de Buenos Aires, Buenos Aires, Argentina; 4 Department of Critical Care Medicine, Sunnybrook Health Sciences Centre Interdepartmental Division of Critical Care, University of Toronto, Toronto, Canada; 5 Department of Critical Care Medicine, Sunnybrook Health Sciences Centre Interdepartmental Division of Critical Care Institute for Clinical Evaluative Sciences, University of Toronto, Toronto, Canada

**Keywords:** Venous thromboembolism, Thromboembolism, Cancer, Pulmonary embolism

## Abstract

**Background:**

Venous thromboembolism (VTE) has been associated with a higher risk of developing malignancy and mortality, and patients with VTE may therefore benefit from increased surveillance. We aimed to construct a clinical predictive score that could classify patients with VTE according to their risk for developing these outcomes.

**Methods:**

Observational cohort study using an existing clinical registry in a tertiary academic teaching hospital in Buenos Aires, Argentina. 1264 adult patients greater than 17 years of age presented new VTE between June 2006 and December 2011 and were included in the registry. We excluded patients with previous or incident cancer, those who died during the first month, and those with less than one year of follow up (< 5%). 540 patients were included. Primary outcome was new cancer diagnosis during one year of follow-up, secondary composite outcome was any new cancer diagnosis or death. The score was developed using a multivariable logistic regression model to predict cancer or death.

**Results:**

During follow-up, one-quarter (26.4%) of patients developed cancer (9.2%) or died (23.7%). Patients with the primary outcome had more comorbidities, were more likely to have previous thromboembolism and less likely to have recent surgery. The final score developed for predicting cancer alone included previous episode of VTE, recent surgery and comorbidity (Charlson comorbidity score), [AUC of 0.75 (95% CI 0.66-0.84) and 0.79 (95% CI 0.63-0.95) in the derivation and validation cohorts, respectively]. The version of this score developed to predict cancer or death included age, albumin level, comorbidity, previous episode of VTE, and recent surgery [AUC = 0.72 (95% CI 0.66-0.78) and 0.71 (95% CI 0.63-0.79) in the derivation and validation cohorts, respectively].

**Conclusions:**

A simple clinical predictive score accurately estimates patients’ risk of developing cancer or death following newly diagnosed VTE. This tool could be used to help reassure low risk patients, or to identify high-risk patients that might benefit from closer surveillance and additional investigations.

**Trial registration:**

ClinicalTrials.gov: NCT01372514.

## Background

Venous thromboembolism (VTE) includes deep vein thrombosis (DVT) or pulmonary embolism (PE) and is an important cause of morbidity and mortality [1]. VTE is also associated with other conditions that influence patient’s mortality prognosis, in particular cancer [2-5]. VTE may complicate the course of a patient with known cancer, but it may also be its first manifestation [6]. According to a systematic review, up to 10% of patients presenting with idiopathic VTE are subsequently diagnosed with cancer during the first year of follow up [7,8]. Moreover, mortality at one year is higher in patients with VTE that develop cancer compared to those that do not [5,9,10].

Suspicion of underlying cancer may lead clinicians to screen for cancer and provide closer surveillance following an acute episode of VTE [7,9,11,12]. However, unselected screening can lead to a higher rate of false positive results, inducing unnecessary anxiety and increasing costs [13]. Conversely, no surveillance after the diagnosis of VTE may delay detection of potentially treatable cancers [8,9]. At present, clinicians typically assess patients’ cancer risk after VTE using conventional approaches to cancer screening that are based on classic risk factors [14-18]. Recent guidelines have proposed specific work up strategies for these patients including computed tomography [19]. However, little evidence exists to help target which individuals should undergo such screening from the entire population of patients with VTE.

We therefore sought to construct a clinical predictive score that could stratify patients according to their risk of subsequent cancer or death. Our overall goal was to identify patients that might benefit from a more intensive screening strategy and surveillance.

## Methods

### Study population

We conducted our study using an institutional registry of 1264 consecutive patients that were admitted between June 2006 and December 2011 to Hospital Italiano, a tertiary teaching hospital in Buenos Aires, Argentina [20]. All adult patients (both inpatients and outpatients, age > 17 years of age) presenting with a new diagnosis of VTE were included in the registry database (Microsoft ACCESS, Redmond, Washington) after providing informed consent. The study protocol was approved by the ethics review board of the Hospital Italiano de Buenos Aires. A full-time research fellow screened all patients at initial diagnosis and updated the database during all follow-up visits. The registry contains information on baseline demographics, clinical history and co-morbidities, physical examination, and laboratory and radiological data. It also contains information on vital status and cancer diagnosis during follow up; cancer diagnosis was ascertained from electronic charts, as a clinical or pathology-based diagnosis. The routine practice at the institution is to continue to follow these patients until death or until they are lost to follow-up. Frequency of follow up and cancer screening was left to the discretion of the individual physicians.

As patients with overt cancer that develop VTE are different from those that present with VTE as a first manifestation of malignancy we excluded patients with cancer diagnosis that preceded VTE, those that were diagnosed with VTE and cancer at the same time (during the same month) and patients who died during the first month following VTE diagnosis. Finally, we excluded those with less than one year of follow up. From the entire sample we created a derivation cohort by randomly selecting two-thirds of the patients, and the remaining third became the validation cohort.

### Model development

We used available variables to construct models that would predict the outcome cancer (primary outcome) and cancer and death (secondary outcome). Death was included as part of the secondary composite outcome as it may act as a competing event regarding the development of cancer. We first selected potentially useful baseline characteristic predictor variables for the multivariable model based on clinical experience and previous literature. Candidate variables included demographic characteristics (age, sex), classic risk factors for thromboembolic disease (major surgery, previous VTE, family history), coexisting illnesses (Charlson comorbidity index score [21]), body mass index (BMI), and laboratory tests (albumin, hemoglobin). We dichotomized continuous variables using their median values as follows: age ≥ 70 years; score on the Charlson comorbidity index ≥ 2; albumin level ≤ 2.5 g/l. Variables were retained only if they remained associated with the primary outcome in a multivariable logistic model using the full model fit [22].

### Score generation

We assigned point scores for each variable in the final model by rounding the corresponding coefficients to integers [23]. We then calculated a total score for every patient by adding the individual points for each risk factor that was present. We calculated sensitivity, specificity, negative and positive predictive values (with 95% confidence intervals) for each cut-off point of the score in order to predict cancer or death at one year [24]. We also calculated negative and positive likelihood ratios (with 95% confidence intervals) [25].

### Validation of the prediction rule

We assessed calibration and discrimination in both the derivation and validation cohorts. Calibration was determined using the Hosmer-Lemeshow test [26] and compared the actual and predicted outcomes within each point stratum for the development and validation cohorts. We evaluated discrimination using receiver operating characteristic curves (ROC) [27]. We compared ROC curves for both cohorts according to the method described by Haney et al. [28].

## Results

The institutional registry contained 1264 patients that were diagnosed with new VTE between June 2006 to December 2011, and complete follow-up information was available on 1211 (95.8%). Of these, we excluded 494 (40.8%) patients who had previously been diagnosed with cancer, 132 (10.9%) who died during the incident hospital admission or during the first month of follow-up, and 45 (3.7%) who were diagnosed with VTE during the last year of the study. A random selection of 349 (two thirds) of the 540 remaining patients comprised the derivation cohort and 191 patients (one third) comprised the validation cohort.

### Patient characteristics

During one-year of follow-up, nearly one-quarter (92; 26.4%, 95% CI 21.4% - 30.6%) of patients died (83; 23.7%, 95% CI 18.5% – 27.4%) or developed cancer (32; 9.2%, 95% CI 18.5% – 27.4%). Lung cancer was the most common diagnosed malignancy (21.9%, 95% CI 7.6% - 36.2%) followed by haematogical disorders (18.7%, 95% CI 5.2% - 32.2%). Nearly one third of patients developed metastatic disease (Additional file 1). Patients with the primary outcome of cancer had more comorbidities and previous VTE, and were less likely to have had recent surgery (Table 1). The patients who developed cancer during follow-up had higher mortality than patients who did not develop cancer (71.9% vs. 18.9%; p < 0.0001) (Table 2).

**Table 1:** Characteristics of patients with versus without cancer at one year

	**Cancer**^**1 **^**(N=32)**	**No cancer (N=317)**	**p value**
Demographic factor			
Age, median (IQR), y	71 (57–81)	73 (57–81)	0.81
Male sex N (%)	18 (56)	125 (39)	0.08
Hospitalized at time of diagnosis N (%)	11 (34)	121 (38)	0.71
Main diagnosis			
DVT diagnosis N (%)	21 (66)	240 (76)	0.21
PE diagnosis N (%)	14 (44)	128 (40)	0.71
VTE risk factors			
Major surgery N (%)	5 (16)	114 (36)	0.02
Previous VTE N (%)	7 (22)	1 (0.3)	<0.001
Family history of VTE N (%)	2 (6.2)	9 (2.8)	0.29
Trauma N (%)	1 (3)	46 (14)	0.08
Oral contraceptives N (%)	0 (0)	13 (4.1)	0.24
Recent travel N (%)	7 (21)	19 (6)	<0.001
Inmovility N (%)	8 (25)	127 (40)	0.09
Comorbidities			
Charlson score, median (IQR)	3 (0.5-4.5)	1(0–2)	<0.001
Physical examination			
BMI, mean (SD), kg/m^2^	26.4 (5)	27.7 (5)	0.24
Laboratory findings			
Albumin levels, median (IQR), mg/dl	3 (IR 2–3.6)	3 (IR 2.6-3.6)	0.61
Hemoglobin, mean (SD), mg/dl	11.3 (IR 2)	11.7 (IR 2)	0.29
Death within one year N (%)	23 (72%)	60 (19%)	<0.001

^1^Cancer within one year.

Abbreviations: *IQR:* interquartile range, *DVT:* deep vein thrombosis, *PE:* pulmonary embolism, *VTE:* venous Thromboembolism, *BMI:* body mass index, *SD:* standard deviation.

Means are compared with t-tests, medians with Mann–Whitney U tests, and dichotomous variables with the chi-square test.

**Table 2:** Characteristics of patients with versus without cancer or death

	**Cancer or Death**^**1 **^**(N=92)**	**No cancer or Death (N=257)**	**p value**
Demographic factor			
Age in years, median (IQR)	75 (61–82)	71 (55–80)	0.04
Male sex, N (%)	46 (50)	97 (37.7)	0.02
Hospitalized at time of diagnosis N (%)	43 (46.7)	89 (34.6)	0.04
Main diagnosis			
DVT diagnosis N (%)	68 (73.9)	193 (75.1)	0.46
PE diagnosis N (%)	36 (39.1)	106 (41.2)	0.41
VTE risk factors			
Major surgery N (%)	21 (22.8)	98 (38.1)	0.005
Previous VTE N (%)	7 (7.6)	1 (0.4)	<0.001
Family history of VTE N (%)	2 (2.2)	9 (3.5)	0.41
Trauma N (%)	9 (9.8)	37 (14.4)	0.17
Oral contraceptives N (%)	1 (1)	12 (4.7)	0.10
Recent travel N (%)	8 (8.7)	18 (7)	0.37
Inmovility N (%)	36 (39)	99 (38)	0.91
Comorbidities			
Charlson score, median (IQR)	2 (0–3)	0 (0–2)	<0.001
Previous stroke N (%)	2 (2.2)	16 (6.2)	0.10
Diabetes N (%)	15 (16.3)	30 (11.7)	0.16
Congestive heart failure N (%)	20 (21.7)	26 (10.1)	0.005
Coronary artery disease N (%)	20 (21.7)	22 (8.6)	0.001
Smoking habit (ever) N (%)	28 (30.4)	89 (34.6)	0.27
Physical examination			
BMI, mean (SD), kg/m^2^	27.9 (5)	26.8 (5)	0.096
Laboratory findings			
Albumin levels mg/dl, median (IQR)	2.9 (IR 2–3.2)	3.2 (IR 2.7-3.6)	0.000
Hemoglobin mg/dl, mean (SD)	11.4 (IR 2)	11.8 (1.8)	0.089

^1^Cancer or Death within one year.

Abbreviations: *IQR:* interquartile range, *DVT:* deep vein thrombosis, *PE:* pulmonary embolism, *VTE:* venous Thromboembolism, *BMI:* body mass index, *SD:* standard deviation.

Means are compared with t-tests, medians with Mann–Whitney U tests, and dichotomous variables with the chi-square test.

### Score development

The multivariable logistic regression model to predict one-year risk of cancer retained the following variables: Charlson comorbidity score, previous VTE, and recent surgery. In the model predicting cancer or death, age and albumin were also retained (Additional file 1). The resulting score values derived from rounding the beta coefficients were the same for both outcomes (Table 3).

**Table 3:** Final scoring systems

	**Cancer**	**Death or cancer**
Previous episode of VTE	3	3
No recent surgery	1	1
Charlson score ≥ 2	1	1
Age ≥ 70 years		1
Albumin ≤ 2.5 mg/dl		2

This scoring system is used to estimate the risk of cancer alone or the combined outcome (cancer or death) at one year after VTE. To derive a final score for each outcome, calculate the sum of values associated with each variable.

### Score performance

We estimated the predicted probability of developing the primary and secondary outcomes using a logistic regression model in both the derivation and validation cohorts (Additional file 1). Hosmer-Lemeshow goodness of fit testing showed good calibration (p=0.65 and p=0.94 in the derivation and validation cohorts, respectively). The final score to predict cancer alone had an AUC of 0.75 (95% CI 0.66-0.84) and 0.79 (95% CI 0.63-0.95) in the derivation and validation cohorts, respectively. The final score to predict the combined outcome of cancer and death had an area under the curve (AUC) of 0.72 (95% CI 0.66-0.78) and 0.71 (95% CI 0.63-0.79) in the derivation and validation cohorts, respectively (ROC curves in Additional file 1). The sensitivities, specificities, positive and negative predictive values, and likelihood ratios associated with each point of the final scores are shown in Table 4 and Table 5.

**Table 4:** Test performance for primary outcome (Cancer)

**Score points**	**Sensitivity (CI 95%)**	**Specificity (CI 95%)**	**NPV (CI 95%)**	**PPV (CI 95%)**	**LLR+ (CI 95%)**	**LLR- (CI 95%)**
**≥** 1	0.97 (0.84-0.99)	0.27 (0.22-0.32)	0.99 (0.93-0.99)	0.11 (0.10-0.12)	1.3 (1.2-1.4)	0.12 (0.017-0.81)
**≥** 2	0.59 (0.42-0.74)	0.78 (0.73-0.85)	0.95 (0.92-0.96)	0.21 (0.14-0.26)	2.7 (1.9-3.8)	0.52 (0.34-0.79)
≥ 4	0.22 (0.11-0.39)	0.99 (0.98-1)	0.92 (0.91-0.92)	0.88 (0.47-0.99)	69 (8.8-545)	0.78 (0.65-0.94)

Abbreviations: *CI: 95%* confidence interval, *LLR+* positive likelihood ratio, *LLR-* negative likelihood ratio, *NPV:* negative predictive value, *PPV:* positive predictive value.

**Table 5:** **Test performance for secondary outcome** (**Death or Cancer**)

**Score points**	**Sensitivity (CI 95%)**	**Specificity (CI 95%)**	**NPV (CI 95%)**	**PPV (CI 95%)**	**LLR+ (CI 95%)**	**LLR- (CI 95%)**
≥1	0.98 (0.92-0.99)	0.14 (0.11-0.19)	0.95 (0.82-0.99)	0.28 (0.27-0.30)	1.1 (1.1-1.2)	0.15 (0.037-0.61)
≥ 2	0.88 (0.80-0.93)	0.44 (0.39-0.51)	0.91 (0.85-0.95)	0.36 (0.33-0.38)	1.5 (1.4-1.8)	0.27 (0.15 -0.47)
≥ 3	0.52 (0.42-0.62)	0.72 (0.66-0.77)	0.81 (0.77-0.84)	0.40 (0.33-0.46)	1.9 (1.4-2.5)	0.66 (0.53-0.83)
≥ 4	0.32 (0.24-0.43)	0.93 (0.89-0.96)	0.79 (0.77-0.81)	0.41 (0.25-0.56)	4.7 (2.7-7.9)	0.73 (0.63-0.84)
≥ 5	0.18 (0.12-0.27)	0.98 (0.97-1)	0.77 (0.75-0.78)	0.85 (0.62-0.96)	15 (4.7-52)	0.82 (0.75-0.91)

Abbreviations: *CI: 95%* confidence interval, *LLR+* positive likelihood ratio, *LLR-* negative likelihood ratio, *NPV:* negative predictive value, *PPV:* positive predictive value.

## Discussion

We developed clinical scores to classify patients according to their risk of cancer, or of cancer and mortality, at one year of follow up after developing a new VTE. The final scores employ common and readily available clinical variables and can be easily calculated at the bedside at the time of VTE diagnosis. In our cohort, the scores had good discrimination and calibration, and could differentiate across a wide range of risks for developing cancer, from only 2% (0 points) to greater than 90% risk (5 points). In addition, our score was able to stratify patients’ cancer or mortality risk from 6% (0 points) to greater than 70 % (6 points or more). These simple scores therefore not only provide important prognostic information but might also be used to identify patients that would benefit from closer surveillance and additional investigations.

The ultimate goal of estimating prognosis is to improve clinical decision-making and thereby improve patient outcomes. Our scores may lead to the diagnosis of some malignancies at an earlier stage, and could therefore result in earlier cancer treatments. In addition, some experts have advocated for alterations to anticoagulation strategies in patients with VTE who also have underlying cancer [29]. Conversely, excluding patients who are at low risk for developing cancer or death from screening strategies and investigations should lead to fewer false positive results, avoid unnecessary treatment strategies, and reduce overall costs. Our score also identifies patients that are at higher risk of death, regardless of their risk of cancer, and this could in turn motivate clinicians to address other conditions such as chronic heart failure or coronary heart disease that might be contributing to this higher mortality risk. We provide an example of potential responses to different score results using hypothetical scenarios in Table 6.

**Table 6:** Possible clinical scenarios and application

**Clinical example**	**Clinical probability and future strategy**
You evaluate a 50 year old man who presented VTE two weeks ago. His is currently under anticoagulant therapy with Warfarin. He presented VTE without any predisposing situation and currently smokes.	The patient´s score = 2. His probability of presenting cancer during the first year of follow up is 17% with a + LLR = 2.6. He could be included into an intensive cancer screening strategy.
You evaluate a 35 year old man after one week of discharge for a thromboembolic event related to a knee surgery. He has no medical history, is otherwise healthy and his albumin levels was 4 mg/dl at admission. His score for the combined outcome is 0.	The patient’s pretest of having the combined outcome at one year is approximately 20%. After the test his probability of having cancer or dying at one year is 6% with a negative predictive value of 93% and negative likelihood ratio of 0.22. The approach could be conservative and diagnostic testing could be withheld.
You evaluate a 73 year old woman who was discharged last week after a deep venous thrombosis of her right lower limb. It is her first event and she doesn’t have any other risk factors. Her albumin levels during hospitalization were 2.3 mg/dl. Otherwise, she is a smoker and has a Charlson score > 2. This patient´s score is 5.	The probability of dying or having cancer at one year is of 60%, PPV of 80 with + LLR of 7. This warrants tight follow up and possibly further diagnostic strategies.

Our study has several strengths. We used a large and comprehensive clinical dataset that was developed specifically to follow consecutive patients with newly diagnosed VTE. The initial evaluation and data collection occurred soon after the VTE diagnosis, increasing the clinical utility of our final scoring system. The loss to follow-up at one year remained very low, decreasing the risk of selection bias. Finally, our cohort includes a large number of patients from across Argentina and from different social backgrounds, increasing the generalizability of our final score.

Our study also has several limitations. We could only evaluate variables that were contained in our database, and it is likely that other clinical variables could increase the predictive accuracy of our score. However, the variables in our final scores are widely available and easily obtained, which should improve the external validity of our model. We included only baseline variables in our model, and were unable to evaluate characteristics that evolve over time and that might further influence a patient’s risk of cancer or death. Our model had high discrimination in both the derivation and validation cohort, similar to that observed for other widely used predictive models [30], but it still will lead to some misclassification of patients. In addition, our validation cohort was derived from the initial sample and was not an independent cohort; it is likely that some loss in discrimination will occur when our scores are applied in other populations. Another limitation is the lack of standardized cancer screening, making it possible that our study is biased by physicians’ decisions to request additional screening tests for patients having the same risk factors identified in our study. However, surveillance using radiological imaging was common throughout the study, with 80% and 34% of patients receiving chest and abdominal computed tomography, respectively, in the first year following VTE diagnosis. Although our scores should help physicians identify patients at higher risk of cancer, it remains unknown whether earlier diagnosis will lead to improved survival [31], especially considering that cancers associated with VTE often have a relatively poor prognosis [6]. Finally, interpreting intermediate risk scores is a challenge common to most predictive models; the optimal approach to surveillance and investigation of these patients is even more uncertain than for those at low or high risk.

## Conclusion

We have developed a simple and clinically relevant score that can predict risk of developing cancer in patients with newly diagnosed VTE. This score could be used to help reassure low risk patients, or to identify high-risk patients that might benefit from increased surveillance and additional investigations. However, our tool should be validated in an externally derived cohort to evaluate its generalizability before it is routinely adopted into clinical practice.

## Abbreviations

VTE: Venous Thromboembolism; ROC curves: Receiver operating characteristic curves; DVT: Deep vein thrombosis; PE: Pulmonary embolism; AUC: Area under the curve; BMI: Body mass index.

## Competing interests

The authors declare that they have no competing interests.

## Authors’ contributions

BLF and FA: concept and study design, data analysis and interpretation, drafting of the manuscript. DG and MLPM: participant recruitment, data acquisition and analysis. FGBQ: participant recruitment, data acquisition and study supervision. FV: participant recruitment, data acquisition, study supervision, critical review of manuscript. AA and DS: data analysis and interpretation, drafting and critical review of manuscript. All authors read and approved the final manuscript.

## Pre-publication history

The pre-publication history for this paper can be accessed here:

http://www.biomedcentral.com/1471-2407/13/352/prepub

## Supplementary Material

Additional file 1 Predictive score for estimating cancer after venous thromboembolism: a cohort study. Click here for file
