# Supplementary material for: Predictive score for estimating cancer after venous thromboembolism: a cohort study
Source: BMC Cancer. 2013 Jul 22;13:352. doi: 10.1186/1471-2407-13-352 (PMC3723428; doi:10.1186/1471-2407-13-352)
Supplement: Additional file 1 — Predictive score for estimating cancer after venous thromboembolism: a cohort study. [file 1471-2407-13-352-S1.docx]

| **Table A1. Multivariate model for the outcome cancer at one year** | | | | | |
| --- | --- | --- | --- | --- | --- |
|  | **Coefficient** | **P value** | **Exp (B)** | **CI 95%** | |
| **No recent surgery** | 1.181 | 0.041 | 3.26 | 1.0 | 10.09 |
| **Previous episode of VTE** | 4.16 | < 0.001 | 64 | 7.07 | 579 |
| **Charlson score ≥ 2** | 1.08 | 0.011 | 2.94 | 1.28 | 6.73 |

Abreviations: VTE, venous tromboembolism

| **Table A3. Calibration for outcome cancer (primary outcome)** | | | |
| --- | --- | --- | --- |
| **ScorePoints** | **Predicted Cancer** | **Observed Cancer** | |
|  |  | **Derivation** | **Validation** |
| **0** | 2% | 1% | 0% |
| **1** | 6% | 7% | 3% |
| **2** | 17% | 15% | 6% |
| **4** | 71% | 67% | 100% |
| **5** | 90% | 100% | 100% |

| **Table A2. Multivariate model for the combined outcome (cancer or death within one year)** | | | | | |
| --- | --- | --- | --- | --- | --- |
|  | **Coefficient** | **P value** | **Exp (B)** | **CI 95%** | |
| **Age ≥ 72 years** | 0.51 | 0.02 | 1.67 | 1.08 | 2.57 |
| **No recent surgery** | 1.04 | 0.001 | 2.83 | 1.50 | 5.40 |
| **Previous episode of VTE** | 2.90 | 0.009 | 18.27 | 2.08 | 159 |
| **Charlson score ≥ 2** | 0.70 | 0.01 | 2.02 | 1.18 | 3.46 |
| **Albumin ≤ 2 mg/dl** | 1.28 | <0.001 | 3.5 | 1.91 | 6.60 |

Abreviations: VTE, venous tromboembolism

| **Table A4. Calibration for secondary outcome (death or cancer)** | | | |
| --- | --- | --- | --- |
| **ScorePoints** | **Predicted Outcome** | **Observed Outcome** | |
|  |  | **Derivation** | **Validation** |
| **0** | 6% | 7% | 0% |
| **1** | 11% | 15% | 18% |
| **2** | 18% | 16% | 20% |
| **3** | 29% | 24% | 26% |
| **4** | 43% | 39% | 50% |
| **5** | 60% | 62% | 53% |
| **6+** | ≥72% | 100% | 100% |

**Figure A1. ROC curve for the outcome Cancer alone at one year.**


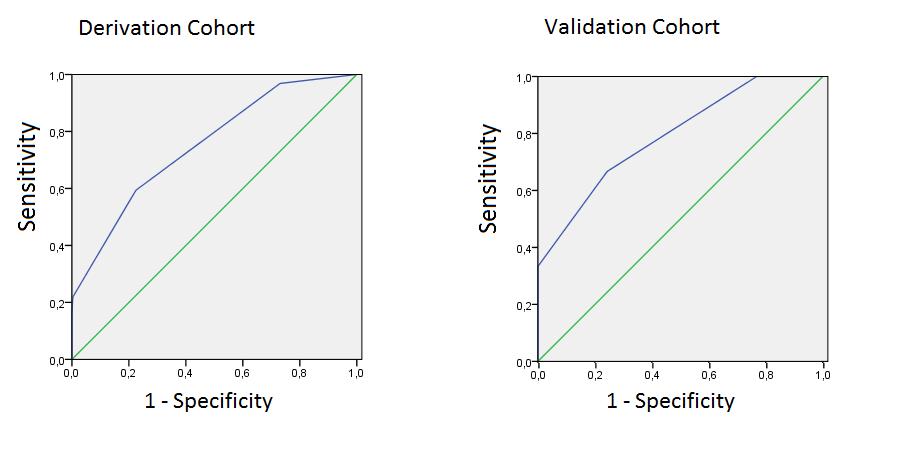


AUC of 0.75 (95% CI 0.66-0.84) and 0.79 (95% CI 0.63-0.95) in the derivation and validation cohorts, respectively

**Figure A2. ROC for the combined Outcome (cancer or death within one year)**


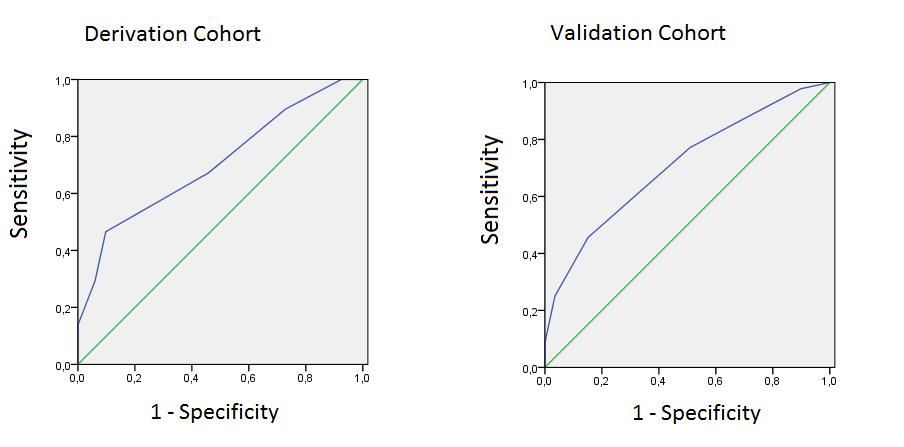


AUC = 0.72 (95% CI 0.66-0.78) and 0.71 (95% CI 0.63-0.79) in the derivation and validation cohorts, respectively.

| Table B1. Cancer diagnosis by primary site | | |
| --- | --- | --- |
| **Type of cancer** | **N (%)** | **95 % CI** |
| **Lung Cancer** | 7 (21.9) | 7.6 – 36.2 |
| **Haematological disorders (Lymphoma-Multiple Myeloma)** | 6 (18.7) | 5.2 – 32.2 |
| **Gynecological tumors** | 6 (18.7) | 5.2 – 32.2 |
| **Renal cell carcinoma** | 4 (12.5) | 1 - 24 |
| **Other** | 4 (12.5) | 1 – 24 |
| ***Metastatic disease*** | 9 (28.1) | 12.5 – 43.7 |
| ***Locally aggressive disease*** | 6 (18.7) | 5.2 – 32.2 |

Gynecological tumors: ovarian cancer (4), breast cancer (1), cervical cancer (1)

Other includes: Primary tumor of unknown origin (2), colorrectal carcinoma (1) or head and neck cancer (1)
